# Assessing efficacy in important subgroups in confirmatory trials: An example using Bayesian dynamic borrowing

**DOI:** 10.1002/pst.2093

**Published:** 2021-01-21

**Authors:** Nicky Best, Robert G. Price, Isabelle J. Pouliquen, Oliver N. Keene

**Affiliations:** ^1^ Department of Biostatistics GlaxoSmithKline Research and Development Brentford UK; ^2^ Department of Clinical Pharmacology Modelling & Simulation GlaxoSmithKline Research and Development Stevenage Herts UK

**Keywords:** Bayesian, borrowing, confirmatory, exacerbation, paediatric, subgroup

## Abstract

Assessment of efficacy in important subgroups – such as those defined by sex, age, race and region – in confirmatory trials is typically performed using separate analysis of the specific subgroup. This ignores relevant information from the complementary subgroup. Bayesian dynamic borrowing uses an informative prior based on analysis of the complementary subgroup and a weak prior distribution centred on a mean of zero to construct a robust mixture prior. This combination of priors allows for dynamic borrowing of prior information; the analysis learns how much of the complementary subgroup prior information to borrow based on the consistency between the subgroup of interest and the complementary subgroup. A tipping point analysis can be carried out to identify how much prior weight needs to be placed on the complementary subgroup component of the robust mixture prior to establish efficacy in the subgroup of interest. An attractive feature of the tipping point analysis is that it enables the evidence from the source subgroup, the evidence from the target subgroup, and the combined evidence to be displayed alongside each other. This method is illustrated with an example trial in severe asthma where efficacy in the adolescent subgroup was assessed using a mixture prior combining an informative prior from the adult data in the same trial with a non‐informative prior.

## 1 INTRODUCTION

Analysis by key subgroups is an important aspect of assessing the results of confirmatory trials. When there is evidence of an overall effect of treatment, there are important questions about whether the effect shown applies to subgroups defined by demographic factors of sex, age and race and whether the effect applies in regions across the world. The need to examine these specific subgroups is described in regulatory guidance, for example, the FDA require summaries of efficacy and safety by demographic subgroups^1^ and for a multi‐regional trial an evaluation of consistency of treatment effects across regions is required by ICH E17.^2^ A related problem is identifying and estimating potentially meaningful treatment effects in subgroups in settings where the estimated overall effect is small. This is due to averaging a large value in a target subgroup and a small value in a much larger complementary subgroup.

An initial approach to such questions is to present results separately by subgroups and this is often supplemented with statistical tests of interaction. Sample size typically limits the ability to convincingly show evidence of a treatment effect when a subgroup is considered in isolation. Interaction tests cannot be used to show consistency of effect since a non‐significant p‐value does not provide evidence of no effect and therefore may not rule out differences in efficacy that are of clinical interest. Formal methods for defining consistency of effect based on requiring a subgroup to show a given level of effect (e.g., that the effect size that is at least positive or that the effect size is at least some percentage of the overall effect) are problematic.^3^

When assessing the evidence for treatment effect in a subgroup, there is clear value in using information from subjects outside the subgroup. These subjects in the same study provide data that is relevant to the assessment of efficacy in the specific subgroup of interest. The use of data from subjects not in the specific subgroup has been referred to as extrapolation.^4^ However, the term ‘extrapolation’ on its own implies use of data only from subjects outside the subgroup; the term partial extrapolation^5^ encompasses the situation described in this article where limited data exists for the subgroup of interest.

One technique for using data from outside the subgroup is Bayesian shrinkage.^6^ In the Empirical Bayes approach,^7^ subgroup estimates are obtained by taking a weighted average of the estimate in the subgroup and the overall effect, with weights determined by the ratio of variability within subgroup to the between subgroup variability. Subgroup estimates are shrunk towards the overall effect. More shrinkage occurs as the *within* subgroup variability increases (i.e., as precision of the subgroup estimates decreases) and/or as heterogeneity *between* subgroups decreases (i.e., as similarity of individual subgroup point estimates increases), and the data decide how much borrowing is done. The Bayesian shrinkage approach therefore depends on these estimates of relative variability.

Fully hierarchical Bayesian shrinkage methods have also been proposed for subgroup estimation.^8^ One challenge with both empirical and hierarchical Bayes shrinkage estimation is to obtain reliable estimates of the between‐subgroup heterogeneity variance when the number of subgroups is small. In this case, inference about the subgroup‐specific treatment effects can be sensitive to assumptions made about the heterogeneity.

## 2 BAYESIAN DYNAMIC BORROWING

Bayesian dynamic borrowing^9^, ^10^ represents a novel approach to the problem of assessing the evidence for efficacy in a specific subgroup, alongside an overall positive effect. In this approach, the results from the complementary subgroup (the ‘source’ subgroup) are used to construct an informative prior for the treatment effect θ in the subgroup of interest (the ‘target’ subgroup). This informative prior is combined with a weak prior distribution, assuming no knowledge of treatment effect in the target subgroup, to construct a robust mixture prior. The motivation for inclusion of the weak prior is to introduce scepticism about the relevance of the data from the source subgroup. This combination of priors allows for dynamic borrowing of prior information; the analysis learns how much of the source subgroup prior information to borrow based on the consistency between the target subgroup and the source subgroup.

The use of a mixture prior in Bayesian dynamic borrowing has some similarities to use of ‘lump‐and‐smear’ (also known as ‘spike‐and‐slab’) priors to do variable selection in regression (e.g., Spiegelhalter et al,^11^ section 5.5.4). However, in the case of dynamic borrowing, the aim is not to evaluate the probability that a true interaction exists and there is no variable selection. When using ‘lump‐and‐smear’ priors, a high probability is placed on a single value of no effect, while here the informative prior represents a range of values reflecting results in the source subgroup.

The robust mixture prior method has an important interpretation as a model averaging technique.^12^ In this context, the informative and weak components of the mixture can be thought of as two alternative models or assumptions about the relationship between the treatment effects in the source and target subgroup:

- Model *M*_source_ bases the prior for the target subgroup treatment effect on the estimated effect in the source subgroup (e.g., by setting the target subgroup prior equal to the posterior distribution of the source subgroup treatment effect). This model represents the belief that the treatment effects in the source and target subgroups are similar; if the source posterior is used directly as the prior, the resultant posterior for the target subgroup under this model will be equivalent to pooling the information from the source and target subgroups.
- Model *M*_weak_ specifies a weak (vague) prior for the target subgroup treatment effect. This model represents the belief that the treatment effects in the target and source subgroups are independent, and hence that the source subgroup provides no relevant information about the target subgroup effect; the resultant posterior for the target subgroup under this model will be based on the data from the target subgroup only.

Each model is given a prior probability, where p(*M*_source_) ∈ (0, 1) is the prior belief that the treatment effects in the source and target subgroups are similar, and p(*M*_weak_) = 1‐p(*M*_source_) is the prior belief that the treatment effects in the source and target subgroups are independent. The robust mixture prior is then equivalent to the marginal prior for the treatment effect θ:

π(θ) = p(*M*_source_) π(θ | *M*_source_) + p(*M*_weak_) π(θ | *M*_weak_).

Given the observed data in the target subgroup, the conditional posteriors under each model are updated separately to give π (θ | *M*_source_, y) and π (θ | *M*_weak_, y). Similarly, the prior model probabilities are updated via Bayes theorem to obtain posterior model probabilities:

(1) $$\;p\left(M_{\text{source}}|\;y\;\right)=\frac{f\left(y\;|\;M_{\text{source}}\right)\;p\left(M_{\text{source}}\right)\;}{f\left(y\;|\;M_{\text{source}}\right)\;\;p\left(M_{\text{source}}\right)+f\left(y\;|\;M_{\text{weak}}\right)\;\;p\left(M_{\text{weak}}\right)}$$

where f (y | *M*_*i*_), *i* ∈ (*source*, *weak*) is the marginal likelihood of the data y under model *M*_*i*_.

Posterior inference about the target subgroup may then proceed by adopting a model averaging approach; the posterior distribution of the target subgroup treatment effect is then a weighted average of the posterior distributions under each model, weighted by their respective posterior model probabilities:

π(θ | y) = p(*M*_source_| y) π(θ | *M*_source_, y) + p(*M*_weak_| y) π(θ | *M*_weak_, y).

The model probabilities p(*M*_source_) and p(*M*_weak_) correspond to the prior weights on the informative and weak components, respectively, in the robust mixture formulation, and for notational convenience we will denote these by *w* and (1 − *w*). Inspection of Equation (1) reveals how the *dynamic* borrowing property of robust mixture priors works. When the mixture prior is combined with the observed data from the target subgroup of interest, *w* is updated according to how consistent the data in the target subgroup are with the source subgroup. The more consistent they are, the larger will be the marginal likelihood of the target subgroup data under the informative prior model, leading to an increase in the posterior weight w^*^ (i.e., the posterior probability for model *M*_source_) relative to the prior weight *w* and hence greater the borrowing from source to target subgroup. Conversely, as prior‐data conflict increases, the marginal likelihood of the target subgroup data under the informative prior model reduces, and when the conflict is sufficiently large it becomes lower than the marginal likelihood of the data under the weak prior (i.e., f (y | *M*_*source*_) < f (y | *M*_*weak*_)). This results in w^*^ being lower than *w*, so that the informative prior is down‐weighted and posterior inference is based more heavily on the observed data in the target subgroup.

The choice of prior probability (weight) to place on the informative component is a subjective judgement that should reflect the scientific plausibility of the similarity assumption. This could be pre‐specified. Alternatively, in a retrospective subgroup analysis, a *tipping point* analysis can be carried out to identify the minimum prior weight (*w* = p(*M*_*source*_)) that needs to be placed on the source subgroup component of the robust mixture prior in order for the estimate of efficacy in the target subgroup to show statistically significant evidence of treatment benefit. The scientific credibility of this tipping point as a lower bound on the strength of prior belief in the similarity assumption can then be assessed. In a Bayesian framework, a posterior probability of at least 97.5% that there is a treatment benefit is a criteria that can be used to represent statistically significant evidence of efficacy.

If the *observed* treatment effects in the two subgroups are very similar, then even a low prior weight on the informative component will be updated to a much higher posterior weight. This will result in the posterior estimate of treatment effect in the target subgroup borrowing strongly from the source subgroup. On the other hand, if there is conflict between the observed subgroup effects, then the posterior weight will not increase as much and may decrease if there is sufficiently strong conflict. Therefore, crucially, the tipping point is not just driven by the gain in precision from borrowing more when there is a higher prior weight, but it also reflects the observed evidence of conflict/consistency between the subgroups in the data.

For computational convenience and ease of generalisation, the data from a given source can be summarised following the approach used by Spiegelhalter et al.^11^ (section 2.4), an approach often used in aggregate‐level meta‐analysis. Specifically, the data likelihood is approximated by a Normal distribution with mean and variance equal to the point estimate and squared standard error of the target parameter (usually the treatment effect on a suitable transformed scale) obtained from a standard regression analysis of the data. This enables the Bayesian analysis to be carried out in two stages: (a) regression analysis is carried out using standard software, and results summarised to generate an approximate Normal likelihood for each data source; (b) the Normal likelihoods can then be used to construct prior and posterior distributions using conjugate Bayesian calculations, as outlined below. Note that use of conjugate likelihoods and priors is not a general requirement of the method however, and the robust mixture prior approach can be implemented using MCMC for non‐conjugate models.

## 3 CASE STUDY USING DYNAMIC BORROWING TO ASSESS A SUBGROUP TREATMENT EFFECT

### 3.1 Trial details

An example of this Bayesian dynamic borrowing modelling approach is provided by a post hoc analysis of the MENSA trial of mepolizumab in severe asthma.^13^ This randomised, placebo‐controlled, double‐blind, parallel group trial compared mepolizumab 100 mg SC (*n* = 194) and mepolizumab 75 mg IV (*n* = 191) with placebo (*n* = 191), given every 4 weeks for 32 weeks in patients with severe asthma with an eosinophilic phenotype who had a history of at least two asthma exacerbations in the previous year while receiving treatment with high dose inhaled steroids and at least 3 months of treatment with an additional controller. The trial was funded by GlaxoSmithKline (ClinicalTrials.gov number: NCT01691521). The primary endpoint was the rate of clinically significant exacerbations, which were defined as worsening of asthma such that the treating physician elected to administer systemic steroids for at least 3 days or the patient visited an emergency department or was hospitalised. Analysis was performed using a negative binomial generalised linear model with a log link function.^14^ The model included a categorical covariate for age group (12–17 years old, ≥18 years old) and the interaction of age group with treatment group, with additional adjustment for baseline covariates (oral corticosteroid [OCS] use, region, exacerbations in the previous year and baseline %predicted FEV_1_).

The trial included 25 adolescent (ages 12–17) and 551 adult subjects (aged ≥18). In the overall population the trial showed strong evidence of a reduction in the rate of exacerbations. The rate of exacerbations was reduced by 47% (95% CI: 28–60) among patients receiving 75 mg IV mepolizumab and by 53% (95% CI: 36–65) among those receiving 100 mg SC mepolizumab, as compared with those receiving placebo. The two active treatment arms provided similar reductions in exacerbation rate compared to placebo and were therefore combined for the evaluation of subgroups; overall the reduction with the two active treatments combined was 50% (95% CI: 35–61).

There was interest in assessing the treatment effect in adolescents but due to the low incidence of severe asthma with an eosinophilic phenotype in adolescents, the conduct of a separate study was considered impractical and there were insufficient adolescent subjects in the MENSA study to show statistical significance when this subgroup was analysed separately. A Bayesian dynamic borrowing approach allowed assessment of the degree of belief needed in the relevance of the adult data to conclude that there was evidence of efficacy in the adolescent subgroup.

A standard Bayesian analysis of the observed efficacy response data for the adolescent subjects would use the adult data as a prior distribution. The resulting posterior estimate of adolescent efficacy is then equivalent to the efficacy estimate based on the original analysis of the full data (i.e., adults and adolescents combined). This therefore assumes that the treatment effect in adolescents is the same as that in adults.

As described above, the standard Bayesian analysis was extended to incorporate a robust mixture prior distribution^9^ which allows for ‘dynamic borrowing’ of prior information – that is, the analysis learns how much of the adult prior information to borrow based on the consistency between the adolescent data and adult prior.

### 3.2 Informative prior component

**FIGURE 1:**
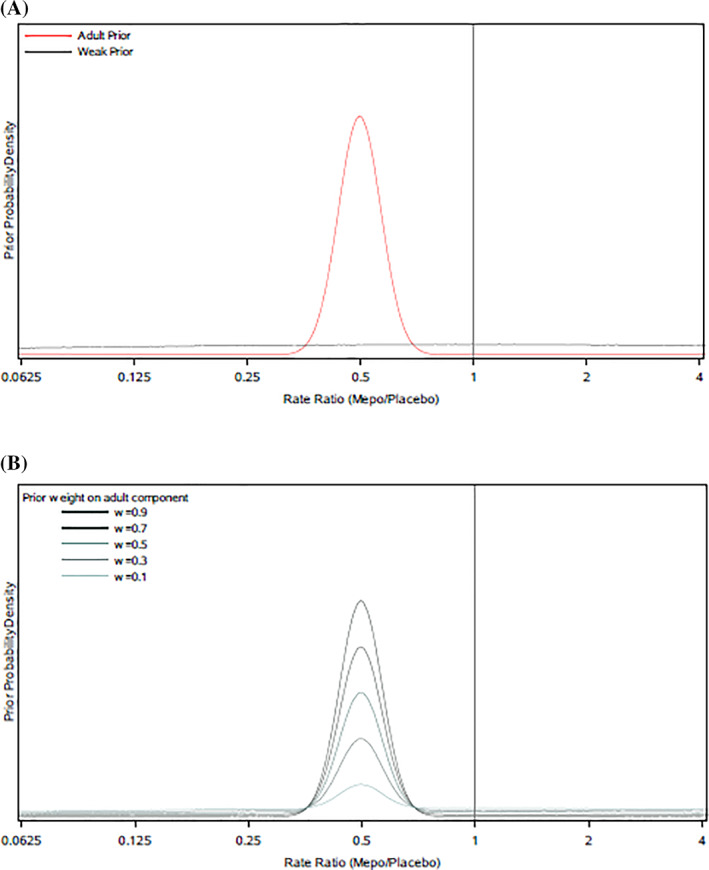
Prior distributions for the adolescent efficacy response: (A) informative (adult) and weak priors, (B) robust mixture prior representing mixture of adult and weak priors, for differing choices of prior weight *w* on adult component

The informative component of the mixture prior was given by the posterior distribution of the log rate ratio of exacerbations on mepolizumab versus placebo estimated from the adult study, assuming an initial vague prior. Using the Normal approximation to the adult data likelihood described above, this is equivalent to a Normal distribution with mean and variance equal to the point estimate and squared standard error of the log rate ratio obtained from negative binomial regression of the observed exacerbation counts in adults. This gave an informative prior component of Normal(mean = −0.694, variance = 0.017; Figure 1A).

### 3.3 Weak prior component

The weak prior was constructed to be a unit‐information normal distribution centred at a mean of zero. This prior represents minimal prior information about the direction or magnitude of the adolescent treatment effect and was included to acknowledge the possibility that there may be differences between adolescents and adults. It is not possible to use a completely flat prior here because the robust mixture must be a so‐called ‘proper’ distribution and have a cumulative probability density of one. A unit‐information prior is designed to be minimally informative; its variance is scaled such that the information content of the prior is approximately equivalent to that provided by a single subject. This variance was determined by taking the squared standard error of the log rate ratio obtained from the adolescent data (which was 0.703^2^) and multiplying it by *N*, where *N* is the total adolescent sample size (Figure 1A). This gave a weak prior component of Normal(mean = 0, variance = 12.4).

### 3.4 Robust mixture prior

The robust mixture prior was defined as a two‐component weighted mixture of the informative adult prior and the weak prior, with weights *w* and (1 − *w*) respectively (Figure 1B). The prior weight, *w*, assigned to the informative prior component represents the prior degree of confidence that the adult data apply to the adolescent population and was varied between 0 and 1 in increments of 0.05.

Robust mixture prior $=w\times \text{Normal}\left(m_{\text{adult}},v_{\text{adult}}\right)+\left(1-w\right)\times \text{Normal}\left(m_{\text{weak}},v_{\text{weak}}\right)$

$\;=w\times \text{Normal}\left(-0.694,0.017\right)+\left(1-w\right)\times \text{Normal}\left(0,12.4\right)$

### 3.5 Posterior distribution for adolescent efficacy

The robust mixture prior was updated with the adolescent data as follows. A normal approximation N(*y*_*adol*_, *v*_*adol*_) was assumed for the adolescent data likelihood for the log rate ratio, where *y*_*adol*_ and *v*_*adol*_ are the point estimate and squared standard error for the log rate ratio of exacerbations on mepolizumab versus placebo obtained from negative binomial regression analysis of the adolescent data. This adolescent data likelihood was combined with the robust mixture prior using standard conjugate Bayesian theory to obtain a posterior mixture distribution for the adolescent treatment effect (log rate ratio):

Posterior mixture for adolescent log rate ratio = $w^{*}\times \text{Normal}\left(m_{\text{adult}}^{*},v_{\text{adult}}^{*}\right)+\left(1-w^{*}\right)\times \text{Normal}\left(m_{\text{weak}}^{*},v_{\text{weak}}^{*.}\right)$

where:

$m_{\text{adult}}^{*}=v_{\text{adult}}^{*}\left(\frac{m_{\text{adult}}}{v_{\text{adult}}}+\frac{y_{\text{adol}}}{v_{\text{adol}}}\right)\;$ and $\frac{1}{v_{\text{adult}}^{*}}=\frac{1}{v_{\text{adult}}}+\frac{1}{v_{\text{adol}}}$

$m_{\text{weak}}^{*}=v_{\text{weak}}^{*}\left(\frac{m_{\text{weak}}}{v_{\text{weak}}}+\frac{y_{\text{adol}}}{v_{\text{adol}}}\right)\;$ and $\frac{1}{v_{\text{weak}}^{*}}=\frac{1}{v_{\text{weak}}}+\frac{1}{v_{\text{adol}}}$

$w^{*}=\frac{C_{\text{adult}}\times w}{C_{\text{adult}}\times w+C_{\text{weak}}\times \left(1-w\right)}$

$C_{\text{adult}}=\frac{\exp\left\{-0.5{\left(y_{\text{adol}}-m_{\text{adul}t}\right)}^{2}/\left(v_{\text{adol}}+v_{\text{adult}}\right)\right\}}{\sqrt{\left(v_{\text{adol}}+v_{\text{adult}}\right)}}$

$C_{\text{weak}}=\frac{\exp\left\{-0.5{\left(y_{\text{adol}}-m_{\text{weak}}\right)}^{2}/\left(v_{\text{adol}}+v_{\text{weak}}\right)\right\}}{\sqrt{\left(v_{\text{adol}}+v_{\text{weak}}\right)}}$

The posterior means and variances of each component are the usual means and variances obtained from conjugate Bayesian updating of that individual prior component, and the posterior weight is a function of the prior weight and coefficients *C*_*adult*_ and *C*_*weak*_ that are proportional to the marginal likelihood of the adolescent data under the adult and weak priors respectively^9^; see also Equation (1).

### 3.6 Tipping point analysis

Based on knowledge of the disease pathology in adults and adolescents and the mechanism of action of mepolizumab, there is a strong rationale to believe that efficacy in adolescents should be consistent with that in adults.

To assess the sensitivity to the strength of prior belief in the consistency assumption, a tipping point analysis was carried out to identify how much prior weight (*w*) needed to be placed on the adult prior component of the robust mixture prior in order for the posterior estimate of efficacy for adolescents to show evidence of treatment benefit. In a Bayesian framework, this was defined as requiring the upper limit of the 95% posterior credible interval for the rate ratio of exacerbation on mepolizumab versus placebo to be below 1 (or equivalently, greater than 97.5% posterior probability that the exacerbation rate is lower on mepolizumab than placebo). As an additional summary of the tipping point analysis, the updated posterior weight (w^*^) assigned to the informative adult prior was plotted against the prior weight (*w*) to identify how much prior confidence in the assumption of similar efficacy was needed in order to result in (a) at least 80%, (b) at least 90%, and (c) at least 95% posterior weight on the adult prior (and hence on the similar efficacy assumption) once the adolescent data was taken into account.

R code for implementing the tipping point analysis is provided in the Supporting Information.

## 4 RESULTS

**FIGURE 2:**
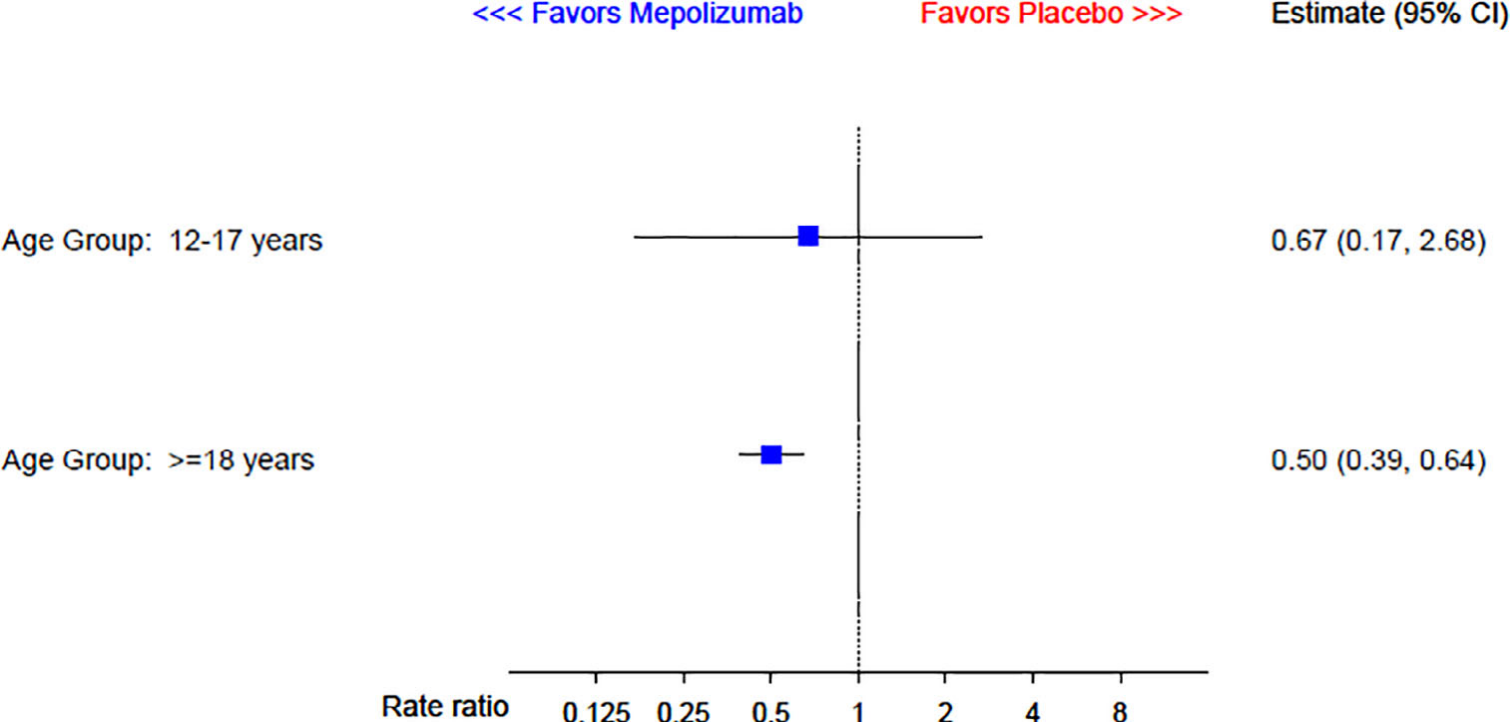
Analysis of rate of clinically significant exacerbations by age group

Results of the separate analysis of adolescent (*n* = 25) and adult (*n* = 551) data are shown in Figure 2. When analysed separately, the point estimate for the relative rate in adolescents is 0.67, corresponding to a 33% reduction in exacerbations on mepolizumab, but the confidence interval is wide (0.17–2.68), reflecting additional uncertainty due to the smaller sample size in the subgroup. On the log rate ratio scale, this yielded an approximate Normal likelihood of Normal(−0.395, 0.730) for the adolescent data.

**FIGURE 3:**
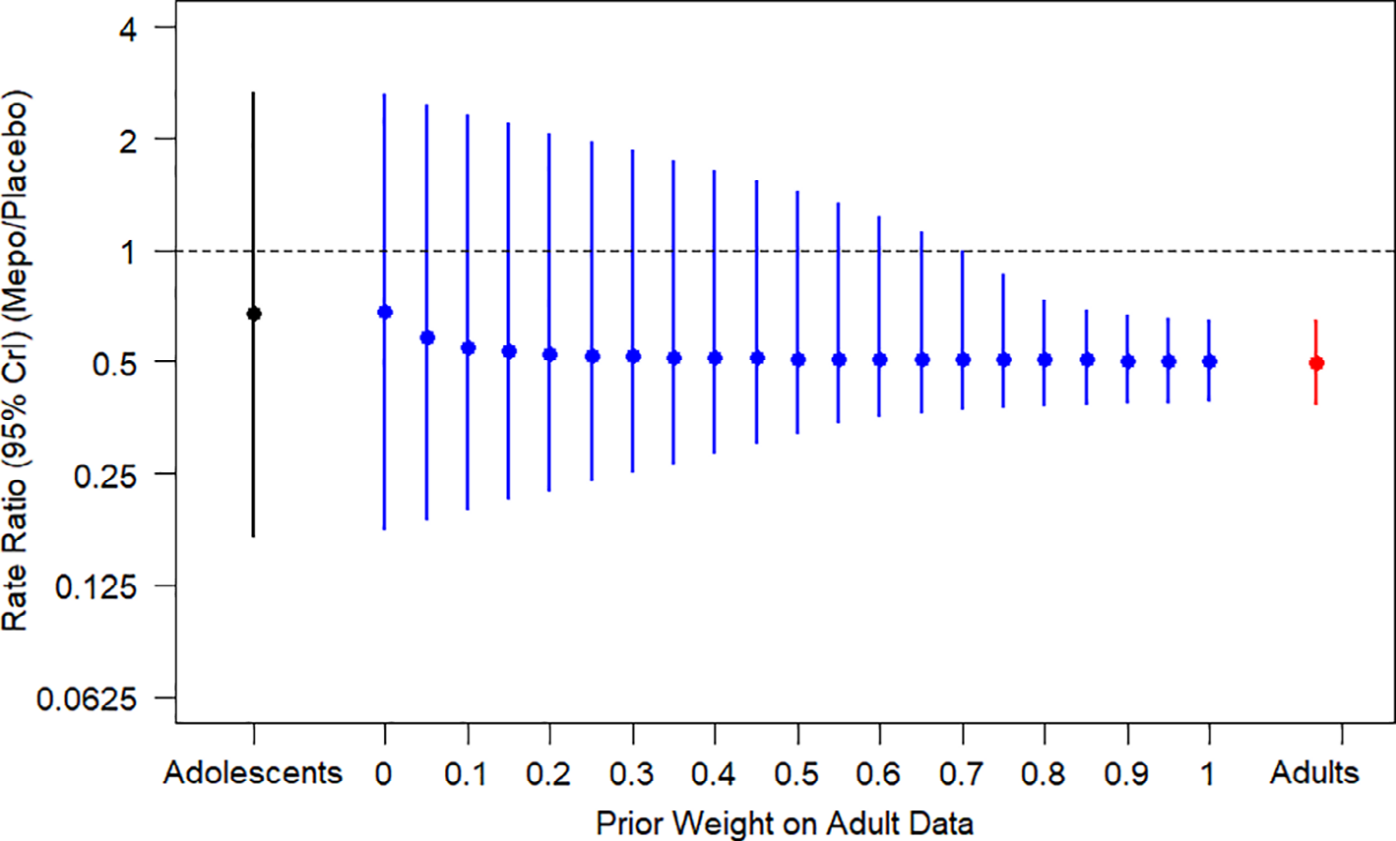
Posterior median and 95% credible interval (CrI) for the estimated rate ratio in adolescents against prior weight given to the adult prior component

Figure 3 presents the posterior medians and 95% credible intervals for the estimated exacerbation rate ratio of mepolizumab versus placebo in adolescents as a function of the prior weight given to the adult component in the robust mixture prior. Note that the adolescent treatment effect estimates based on prior weights of 0 and 1 correspond to standard conjugate Bayesian analyses of the adolescent data using either the weak prior alone, or the adult prior alone, respectively.

Figure 3 shows that the tipping point occurs at a prior weight between 0.65 and 0.7 – that is, for prior weights of 0.7 or higher the upper limit of the posterior 95% credible interval for the rate ratio is below one, indicating a statistically significant treatment benefit of mepolizumab in adolescents. For example, assuming a prior weight of 0.7 gives posterior median and 95% credible interval for the relative rate in adolescents of 0.51 (0.37, 0.99; Table 1).

**TABLE 1:** Posterior median and 95% credible interval (CrI) for the estimated Rate Ratio of exacerbations in adolescents against prior weight given to the adult prior component

Prior weight on adult data	Rate Ratio (95% CrI) (Mepo/Placebo)
0.00	0.68 (0.18, 2.64)
0.10	0.55 (0.20, 2.33)
0.20	0.53 (0.23, 2.07)
0.30	0.52 (0.25, 1.85)
0.40	0.51 (0.28, 1.64)
0.50	0.51 (0.32, 1.44)
0.60	0.51 (0.36, 1.23)
**0.70**	**0.51 (0.37, 0.99)**
0.80	0.51 (0.38, 0.74)
0.90	0.51 (0.39, 0.67)
1.00	0.50 (0.39, 0.65)

*Note*: Bold value of 0.70 for prior weight indicates smallest value of prior weight for which the credible interval excludes 1.

**FIGURE 4:**
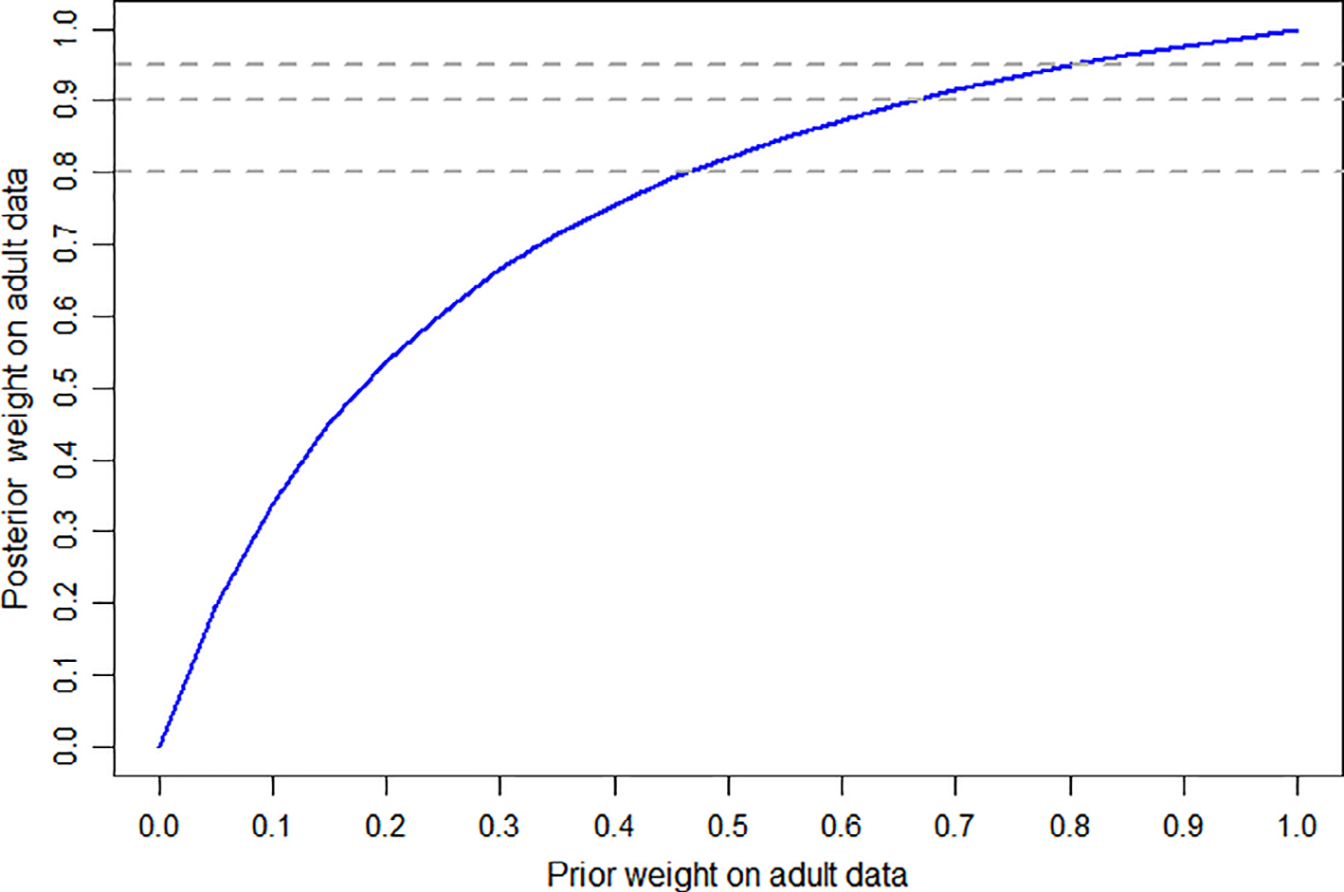
Prior versus posterior weight on adult component of the robust mixture prior

Figure 4 presents the posterior weight assigned to the adult information after accounting for the adolescent data, plotted against the prior weight given to the adult component in the robust mixture prior. Various threshold posterior weights are marked with horizontal reference lines. For example, if a value of around 0.45/0.65/0.8 is considered credible for the prior probability of similarity between adult and adolescent efficacy, then the observed concordance between the adult and adolescent data is sufficient to increase this to a posterior probability of around 0.8/0.9/0.95 respectively.

**FIGURE 5:**
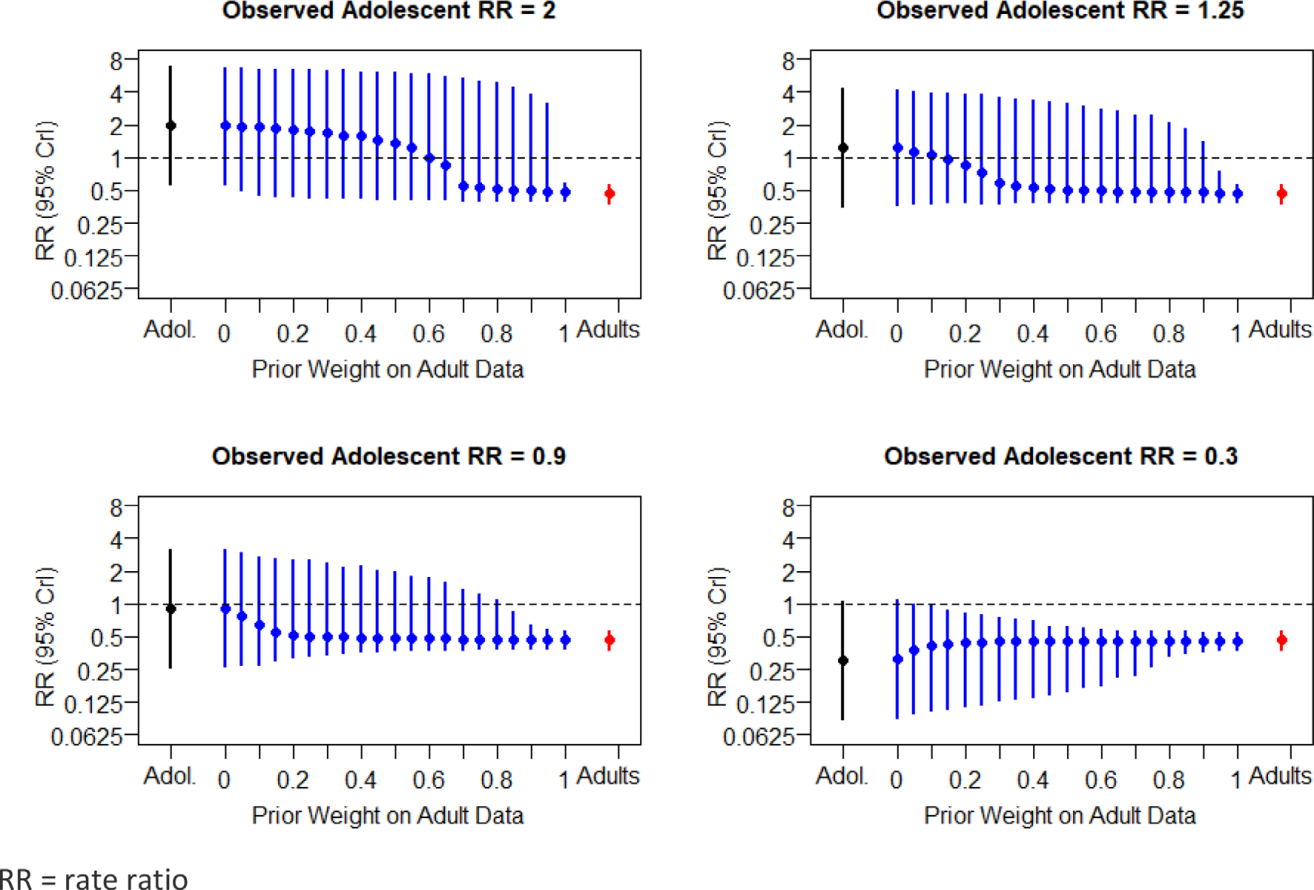
Hypothetical examples with different levels of conflict between subgroups

In the mepolizumab example, observed reductions in exacerbations were similar between the adolescent subgroup and the adult subgroup. In order to investigate the robustness of the Bayesian borrowing method, alternative hypothetical assumptions were made on the size of the effect in the adolescent subgroup to represent greater amounts of observed conflict between the adolescent and adult subgroup effects. The assumed hypothetical observed relative rates (active/placebo) were set to be 2.0, 1.25, 0.9 and 0.3 – values which lie near to, or outside of, the 95% confidence limits for the adult treatment effect. The behaviour of the prior and posterior weights, and the corresponding tipping point under these different scenarios for the source and target subgroup effects are illustrated in Figure 5. If the observed adolescent relative rate had been 2.0 or 1.25 (i.e., an observed result in favour of placebo), then a prior weight of 1.0 or 0.95 respectively would be required to confirm evidence of efficacy. That is, in these hypothetical cases, the adolescent data would not have supported a conclusion of efficacy unless prior certainty, or near certainty, in the similarity of adult and adolescent treatment effects could be justified. This provides reassurance that the method does distinguish cases of subgroups where additional information is needed.

## 5 DISCUSSION

Examination of results of confirmatory trials by subgroup is increasingly emphasised. There is often imbalance in the numbers of patients in each subgroup and when separate analysis is conducted for each subgroup, small numbers of patients leads to large variability as reflected in wide confidence intervals for the observed effect. Importantly, trials are typically designed to demonstrate significant treatment effects in the overall population and not within each individual subgroup.

When there is a requirement to assess evidence of efficacy in a specific subgroup, Bayesian dynamic borrowing can be a useful approach. In the example presented for the assessment of efficacy in an adolescent subgroup, the robust mixture prior combines an informative prior based on adult data with an uninformative prior assuming no knowledge of treatment effect. This robust mixture prior methodology reduces the impact of an over‐optimistic informative prior and down‐weights or discounts this prior information in the case of increasing prior‐data conflict. In the case of disagreement between the observed data (likelihood) and specified mixture prior, the less‐favoured component of the mixture prior is wholly or partially rejected as the conflict becomes increasingly extreme. In this case study the available efficacy data within adolescent subjects appear consistent with that observed in adults, with increases seen in the updated posterior weight attached to the informative adult prior once the adolescent data were considered.

A key challenge with the use of Bayesian dynamic borrowing is the determination of the choice of prior weight for the informative prior. One approach to overcome this is to use a range of weights and determine the tipping point at which there is a posterior probability of >97.5% that the effect of treatment is greater than zero. The more prior weight required, the greater the amount of informative prior information that must be extrapolated before evidence of an effect can be formally concluded. The case study above considered the full range of possible beliefs (prior weights *w* ranging from 0 to 100%) regarding the relevance of the adult data when estimating the effect in adolescents. It showed that if we are willing to assume prior odds of at least 2.5 to 1 in favour of the adolescent treatment effect being consistent with the adult treatment effect, then, when this judgement is combined with the adolescent trial data, we can conclude there is evidence of a reduction in exacerbations in adolescents.

The tipping point approach we have presented can be seen as a type of ‘analysis of credibility’ or ‘reverse‐Bayes’ method. The aim of these approaches is to allow the extraction of the properties of the prior distribution needed to achieve a certain posterior statement for the data at hand. Such approaches are increasingly used to assess the plausibility of scientific claims and findings.^15^ In particular, the approach described here closely mirrors the method of Matthews.^16^ He proposed assessing the credibility of a statistically significant finding by determining how sceptical the prior distribution for the effect of interest would need to be such that, when combined with the data from the study in question, it results in a posterior distribution that is just non‐significant at level *α*, that is, the 100(1‐*α*)% posterior credible interval just covers the null value. Decision‐makers can then assess whether such a sceptical belief is scientifically reasonable, and hence whether or not a fair‐minded sceptic would consider the finding of a statistically significant treatment effect based on the study in question to be scientifically credible. Our approach follows a similar logic. The weight corresponding to the tipping point represents the minimum prior belief in the relevance of the source data needed to find the evidence from the target subgroup data convincing. Any prior belief more sceptical than this would not be able to conclude a positive treatment effect in the target population on the basis of the evidence from the target subgroup data. This tipping point approach thus allows a range of decision‐makers, who may hold different prior beliefs, to assess the credibility of the evidence in the target subgroup. If the evidence is sufficient to convince even a reasonable sceptic, then the finding of a subgroup treatment effect may be considered robust.

An attractive feature of the tipping point analysis, whether conducted post‐hoc or as part of a pre‐planned sensitivity analysis, is that it enables the evidence from the source subgroup, the evidence from the target subgroup, and the combined evidence to be displayed alongside each other. This facilitates a visual, as well as quantitative, assessment of the consistency of these pieces of information, and satisfies an important principle in the regulatory assessment of clinical trials articulated by Weber et al,^17^ which is ‘to assess each piece of information separately before combining the independent pieces for decision‐making’.

Simon and co‐authors^18^, ^19^ have also described a Bayesian approach to subgroup analyses based on averaging the effect in the target subgroup with the effect in the complementary subgroup. The two components are weighted by the a priori estimate of the likelihood of qualitative treatment by subgroup interaction. However, this is achieved by placing a prior distribution on the variance of treatment by subgroup interaction. The weight used depends on the sample size^19^ and the weight is not updated by the observed data.

The example given here was a post hoc analysis of subgroup data collected as part of the overall study. The method can also be applied to a prospectively designed trial in a subgroup where potential exists to borrow data from another external source. An example of successful regulatory use of the method is the FDA approval of belimumab in children with systemic lupus erythematosus.^20^ The approval was supported by a randomised, controlled trial (NCT01649765) that evaluated belimumab vs placebo in 93 paediatric patients. Determination of efficacy was supported by Bayesian borrowing of the established efficacy of belimumab from two phase III adult studies. Pre‐specifying the prior weight for the primary analysis adds credibility to the analysis, even if a tipping point analysis is also conducted as a sensitivity analysis.

One potential concern is whether use of Bayesian borrowing results in an increase in type I error. If the type I error is defined by considering only the sampling distribution of the observed results from the target subgroup under the null hypothesis, then use of additional information from the source subgroup notionally increases this type I error. However, as Campbell observes, ‘if the prior data makes the null hypothesis more unlikely, it may be no surprise that the type 1 error probability calculated under the unlikely null hypothesis is inflated… Then extremely stringent type 1 error probability control does not make as much sense since there is already evidence that the null is not true’.^21^

A more reasonable definition of type I error is based on considering the target subgroup data and the prior (source subgroup) data together. Then, provided the weight applied to the source data is justified, there is no increase in the type I error of incorrectly concluding an effect in the subgroup when none exists.^22^

An alternative is to consider the average type 1 error rate of a Bayesian borrowing design, in which the usual frequentist type 1 error is integrated with respect to a ‘null prior distribution’ for the treatment effect (for example, the source subgroup prior density conditioned on the null hypothesis being true).^23^ Further experience of the practical application of average type 1 error is needed.

A paediatric development plan is mandatory in the United States and in the EU for a new medicine and clinical studies are generally expected unless the disease only affects the adult population.^24^, ^25^ Placebo controlled efficacy studies in a vulnerable population such as paediatrics are difficult to conduct since recruitment is typically challenging and minimising the requirements of clinical trial participation is highly desirable from a patient perspective. When efficacy has been demonstrated in adults and the disease is similar between the adult and paediatric populations, then it is important to incorporate this knowledge from adults in the assessment of efficacy in paediatrics. In this situation, the Bayesian dynamic borrowing approach provides an appropriate method to evaluate evidence of effect in a paediatric population. A similar Bayesian approach to incorporating adult clinical data into paediatric clinical trials has been advocated by Ye and Travis.^26^

Further subgroups that may be suitable for use of this dynamic borrowing approach include those subgroups of specific regulatory interest, for example, sex, race, region. For these subgroups, it is often required to show evidence of effect alongside an overall positive effect. A separate analysis of the subgroup in question does not take account of the information on the effects of treatment in the complementary subgroup. A Bayesian statistical approach is one natural quantitative method to explicitly borrow information from the complementary subgroup to provide inferences on the subgroup under evaluation.

## DISCLOSURE

Funding for the MENSA study was provided by GSK (NCT01691521; 115,588). N.B., R.P., I.P. and O.K. are all GSK employees hold shares in the company.

## Supporting information


**Appendix S1**: Supporting information

## Data Availability

Data sharing is not applicable to this article as no new data were created or analyzed in this study.
