# Supplementary material for: Assessing efficacy in important subgroups in confirmatory trials: An example using Bayesian dynamic borrowing
Source: Pharm Stat. 2021 Jan 21;20(3):551–62. doi: 10.1002/pst.2093 (PMC8247867; doi:10.1002/pst.2093)
Supplement: Supplementary file 1 — Appendix S1: Supporting information [file PST-20-551-s001.docx]

**Supplementary Material**

**#####################################################################**

**#**

**# R code to implement Robust Mixutre Prior Tipping Point analysis**

**# for paper "Assessing efficacy in important subgroups in**

**# confirmatory trials – an example using Bayesian dynamic borrowing"**

**# by Nicky Best, Robert G. Price, Isabelle Pouliquen, Oliver N. Keene**

**#**

**#####################################################################**

**library(RBesT)**

**######################################################################**

**# Read in point estimates and SE of adult and adolescent log rate ratios**

**# (from separate negative binomial regression analysis on MENSA study data in SAS)**

**######################################################################**

**# Adolescents**

**logRR.adol <- -0.3945**

**SE.logRR.adol <- 0.7033**

**# total number of adolescents**

**N.adol <- 25**

**# Adults**

**logRR.adult <- -0.6941**

**SE.logRR.adult <- 0.1303**

**# total number of adults**

**N.adult <- 551**

**# Reference sd (set to be the adolescent SE for a single subject)**

**sigma.ref <- sqrt(N.adol)*SE.logRR.adol**

**#############################**

**# Run tipping point analyses**

**#############################**

**# Set up arrays to store output of tipping point analyses**

**weights <- seq(0,1,by=0.1)**

**M <- length(weights)**

**post.weight <- array(dim=c(M,2))**

**post.mean <- array(dim=c(M,2))**

**post.sd <- array(dim=c(M,2))**

**post.ci <- array(dim=c(M,3))**

**dimnames(post.weight) <- list(paste("w=", weights, sep=""), c("adult", "weak"))**

**dimnames(post.mean) <- list(paste("w=", weights, sep=""), c("adult", "weak"))**

**dimnames(post.sd) <- list(paste("w=", weights, sep=""), c("adult", "weak"))**

**dimnames(post.ci) <- list(paste("w=", weights, sep=""), c("median", "2.5%", "97.5%"))**

**for(i in 1:M) { # loop over different prior weights**

**w <- weights[i] # prior weight on adult component**

**# robust mixture prior**

**adult.prior <- mixnorm(adult=c(1, logRR.adult, SE.logRR.adult), sigma=sigma.ref)**

**robust.mix.prior <- robustify(adult.prior, weight=(1-w), m=0, n=1)**

**# calculate posterior**

**adol.posterior <- postmix(robust.mix.prior, m=logRR.adol, se=SE.logRR.adol)**

**post.weight[i,] <- adol.posterior[1,]**

**post.mean[i,] <- adol.posterior[2,]**

**post.sd[i,] <- adol.posterior[3,]**

**post.ci[i,] <- qmix(adol.posterior, c(0.5, 0.025, 0.975))**

**}**

**# View table of point estimates and 95% CrI for adolescent RR by prior weight**

**round(exp(post.ci),2)**
